# Supplementary material for: Evaluation of the Universal Salt Iodization (USI) surveillance system in Tanzania, 2022
Source: PLoS One. 2024 Apr 19;19(4):e0299025. doi: 10.1371/journal.pone.0299025 (PMC11029623; doi:10.1371/journal.pone.0299025)
Supplement: Attachment — Submitted filename: reviewer comment_May_2023_PLOS.docx [file pone.0299025.s002.docx]

28/05/2023

Dear Editor,

Herewith I have provided my comments.

Thanking you.

Dr Renuka Jayatissa.

Overall, it is very useful data on surveillance. Surveillance data is lacking and currently focused on that subject to establish systems at country level.

There are some abbreviations, e.g TFNC, need to spell out. All the abbreviations should be spelled out in the first place. I felt this is a routine mandatory system in Tanzania as the regulation. If it is considered as a surveillance system, what are the areas included in addition to the mandatory regulation. Health officers’ assessment is part of the regulation, which has been set up with legal action. School samples with nutritionist may be the other set up. Clarity to be improved. Difficult to understand the real surveillance system. This study need to restructure.

**Introduction**

1. Need more background information on Tanzania iodine status after the iodisation in 1990. Rate of goitre improvement, urinary iodine excretion and any available data on current status to justify the surveillance system in the country. Suggest adding a small paragraph for reader to get a clear idea.

**Methods:**

2. Need to provide details of indicators use for each attribute, which will help readers to adopt similar systems.
3. Better to explain the data collected at each level in a summary format. Methodology is not clear.

**Results**

4. Operation of the USI surveillance system – Some of the facts better to be placed in the method section to identify the existence system in the country, which very confusing.
5. Need to display the results according to the tables and figures, very difficult to identify some information. Flow of the information needs to be adjusted in line with tables.
6. Information in tables are also not clear, clarity to be improved.
7. Figure 4.1, better to explain the reason for reduction in different quarters.
8. Results need to be categorized properly to understand readers.

**Discussion**

9. Need to be improved. Surveillance system needs not only iodised salt, urinary iodine excretion levels also. It may be the limitation in this study. These parts need to be included in the discussion. This study is an evaluation. There are key components in evaluation as specified in the method section, which should be discussed in line with results and other literature.

**Recommendation**

10. Some are not relevant to the study, need to restructure.

**Reference**

11. CDC IMRA reference is not included.
