# Supplementary material for: Evaluation of the Universal Salt Iodization (USI) surveillance system in Tanzania, 2022
Source: PLoS One. 2024 Apr 19;19(4):e0299025. doi: 10.1371/journal.pone.0299025 (PMC11029623; doi:10.1371/journal.pone.0299025)
Supplement: Attachment — Submitted filename: reviewer comment_May_2023_PLOS.-Mchau. docx.docx [file pone.0299025.s003.docx]

Dear Editor, please find our responses towards reviewer’s comments, they are really helpful.

**Reviewer #1**

Overall, it is very useful data on surveillance. Surveillance data is lacking and currently focused on that subject to establish systems at country level.

There are some abbreviations, e.g. TFNC, need to spell out. All the abbreviations should be spelled out in the first place. I felt this is a routine mandatory system in Tanzania as the regulation. If it is considered as a surveillance system, what are the areas included in addition to the mandatory regulation. Health officers’ assessment is part of the regulation, which has been set up with legal action. School samples with nutritionist may be the other set up. Clarity to be improved. Difficult to understand the real surveillance system. This study need to restructure.

***Response:*** *Thank you sincerely for your invaluable input and thoughtful suggestions. Your feedback has been instrumental in enhancing our manuscript significantly. We took your comments to heart and made substantial improvements. In particular, we ensured that all abbreviations were spelled out clearly from the outset. Additionally, we have incorporated the requisite regulations of the USI surveillance system, and we have strived to enhance overall clarity to facilitate better comprehension. Your input has been greatly appreciated and has played a pivotal role in strengthening our work.*

**Introduction**

1. Need more background information on Tanzania iodine status after the iodisation in 1990. Rate of goitre improvement, urinary iodine excretion and any available data on current status to justify the surveillance system in the country. Suggest adding a small paragraph for reader to get a clear idea.

***Response:*** *We greatly appreciate your kind suggestion. As per your guidance, we have duly updated the information as needed.*

**Methods:**

2. Need to provide details of indicators use for each attribute, which will help readers to adopt similar systems.

*Response:*  *Thank you for your suggestion. We have made the necessary improvements and incorporated the suggested indicators for each attribute.*

3. Better to explain the data collected at each level in a summary format. Methodology is not clear.

*Response: Thank you; we have enhanced the methodology, and we've also ensured that the data collected at each level has been comprehensively included.*

**Results**

4. Operation of the USI surveillance system – Some of the facts better to be placed in the method section to identify the existence system in the country, which very confusing.

*Response:*  *Thank you for this significant suggestion. In the results section, we have provided a thorough explanation for our decision to include the operation of the USI system, offering a clear rationale for its placement in this part of the document.*

5. Need to display the results according to the tables and figures, very difficult to identify some information. Flow of the information needs to be adjusted in line with tables.

*Response:*  *Certainly, thank you. We have diligently worked on this.*

6. Information in tables are also not clear, clarity to be improved.

*Response: Clarity is well improved, thank you for the nice observation.*

7. Figure 4.1, better to explain the reason for reduction in different quarters.

*Response: It is well explained in the current version, thank you*

8. Results need to be categorized properly to understand readers.

*Response: Thank you so much, we have worked on this and the current version has well organized results.*

**Discussion**

9. Need to be improved. Surveillance system needs not only iodised salt, urinary iodine excretion levels also. It may be the limitation in this study. These parts need to be included in the discussion. This study is an evaluation. There are key components in evaluation as specified in the method section, which should be discussed in line with results and other literature.

*Response: The discussion section has seen substantial improvements, and all specific suggestions have been successfully incorporated. We are particularly pleased with your excellent suggestion, especially considering our system's inability to capture iodized salt, which we have acknowledged as a limitation. Thank you for your valuable input.*

**Recommendation**

10. Some are not relevant to the study, need to restructure.

*Response:*  *Understood. We have thoroughly reviewed all recommendations and retained only those that are pertinent and necessary for each respective entity responsible for the USI surveillance system to understand their role and responsibilities.*

**Reference**

11. CDC IMRA reference is not included.

*Response:*  *Your reminder is greatly appreciated, and we have duly incorporated it into the current draft. Your input has been incredibly helpful, and many of your comments were not only valuable but also essential in enhancing the quality of our manuscript. Thank you very much for your assistance.*

**Reviewer #2:**

**Abstract**

1. The abstract does not clearly state the aim of the study; neither does it adequately describe the methods used. Because there are no aims, the conclusions cannot be properly assessed. Results in the abstract is confusing, could the authors use percentages instead of fractions

*Response: Thank you so much for your very constructive input and suggestions. We have improved methods section by including results and conclusion*

**Introduction**

2. In the section 'Population under study', I do not understand what the authors mean. Can they clarify?

*Response: Thank you for the nice suggestion. We have updated the information as required.*

**Methods:**

3. The methods do not clearly describe the process in my opinion. '

*Response: Thank you for this suggestion, we have improved our methodology*

**Results**

4. In the first 6 paragraphs of the results, the authors keep describing the methods for data collection etc. In my opinion, the results of the study start from ''key findings...

*Response: Thanks for this important suggestion. We have explained well in the results part why we have decided to have operation of the USI system at the result section. We have also rearranged our results section to make easier understanding for the readers*

**Discussion**

5. Discussion: The authors do a good job by explaining the results however, they fail to make a comparative analysis of their results with other studies. Authors should adequately compare their results to other surveillance in other areas across the world or even within Tanzania.

*Response: The discussion part has been well improved and all specific suggestions are incorporated.* *We are happy about your wonderful suggestion.*

**Limitations:**

6. There seem to be so many limitations to this work-lack of generalizability of the results due to Tanzania, the qualitative nature of the work among others. Authors should take time to work on the limitation. Authors also fail to state the strengths of this work.

*Response: We have worked on limitations and we have also included strength of our study*

**Recommendation**

7. The authors state recommendations, and again I am not sure who the recommendations are addressed to. If they can be specific, it would be very helpful. The entire manuscript does not have a conclusion. Can authors include that?

*Response: Okay, we have revised all recommendations and remain with recommendations that are relevant and needed from each respective entity responsible for USI surveillance system to operate to know their responsibility. We have also included conclusion as suggested and thank you so much for this wonderful suggestion.*

Herewith I have provided our response to each of reviewers comments.

Thanking you.

**Dr. Geofrey Mchau.**

**15.10.2023**
