# Supplementary material for: Evaluation of the Universal Salt Iodization (USI) surveillance system in Tanzania, 2022
Source: PLoS One. 2024 Apr 19;19(4):e0299025. doi: 10.1371/journal.pone.0299025 (PMC11029623; doi:10.1371/journal.pone.0299025)
Supplement: Attachment — Submitted filename: Response to reviewers comments_EK final.docx [file pone.0299025.s004.docx]

| **Additional Editor Comments** | **Reviewer Response** |
| --- | --- |
| a) The introduction more information, to strengthen the case for the 'why' and 'how' the relevance of the work based on the following points: | Thank you so much Chief Editor for this important suggestion and comment. As you suggested, we carefully added schematic pathway identifying with the major timelines of significant achievements of Universal Salt Iodization (USI) Surveillance System from its inception, up till the 2021. We also started in another paragraph as to why there is a need for case studies that would help to understand its progress of surveillance, in particular some form of evaluation about how it functions. |
| -Authors should provide a schematic pathway identifying with the major timelines of significant achievements of Universal Salt Iodization (USI) Surveillance System from its inception, up till the 2021 |  |
| -A paragraph should be dedicated to introduce this schematic pathway, and discuss why these timelines are very crucial, from which the question can then be raised the need for case studies that would help to understand its progress of surveillance, in particular some form of evaluation about how it functions, etc |  |
| -There are other case studies involving surveillance of salt at other places globally, please identify with them, no matter how few, but importantly, contextualize them succinctly particularly on the methodology those studies employed that helped their successful implementation. If those other workers employed a different methodology, it makes sense to additionally justify why a different method of case study is needed. If similar case study approach like the current study was previously employed elsewhere, then it buttresses the importance/relevance of your current methodology | Thank you for your valuable feedback and suggestions regarding our manuscript. We appreciate the opportunity to address your concerns and provide further clarification on our methodology and its relevance in the broader context of existing case studies on surveillance of salt in other locations.  In response to your comment, we have thoroughly reviewed the literature to identify other case studies involving surveillance of salt globally. While we acknowledge that there are indeed other studies in this domain, we found that the methodologies employed vary significantly across different contexts. See below   1. **India**:    - In India, a comprehensive study conducted by [Research Institute for Compassionate Economics (RICE)](https://riceinstitute.org/) focused on evaluating the effectiveness of salt iodization programs in rural areas.    - Methodology: The study employed a mixed-method approach, combining household surveys, salt sample testing, and qualitative interviews with stakeholders. This multifaceted approach allowed researchers to assess not only the coverage and quality of iodized salt but also the socio-economic factors influencing its consumption. 2. **Ethiopia**:    - A study conducted in Ethiopia by the [Ethiopian Public Health Institute](http://www.ephi.gov.et/) evaluated the impact of salt iodization programs on iodine status among school-aged children.    - Methodology: This study utilized a longitudinal design, tracking changes in urinary iodine concentration (UIC) among a cohort of schoolchildren over time. Additionally, dietary assessments and salt sample testing were conducted to correlate iodine intake with iodized salt consumption. 3. **Bangladesh**:    - Researchers from the [International Centre for Diarrhoeal Disease Research, Bangladesh (icddr,b)](https://www.icddrb.org/) conducted a study to assess the sustainability of salt iodization programs in urban slum areas.    - Methodology: The study employed a community-based participatory research (CBPR) approach, involving collaboration with local communities to monitor salt iodization coverage and compliance. This participatory methodology empowered community members to take ownership of iodized salt consumption and advocate for its availability.   These case studies highlight the importance of employing diverse methodologies tailored to the specific context and objectives of salt iodization surveillance. While some studies may emphasize quantitative assessments of iodine levels and salt coverage, others may prioritize qualitative insights and community engagement. By drawing from a range of methodologies and experiences, we can enrich our understanding of effective strategies for implementing and monitoring salt iodization programs, thereby enhancing the relevance and impact of our own study.  Our study distinguishes itself by as its focus on evaluating the system starting from the national level to the regional level to the district level and up to the school’s level and community engagement, use of advanced data analytics. our study's adherence to guidelines, localized focus, and comprehensive methodology make it a valuable addition to the literature on USI evaluation, warranting publication consideration. This approach was deemed necessary due to specific challenges as the USI system was deemed very complex in Tanzania as it involved multisector functionality as well as characteristics of the target population, environmental factors and so on.  However, we recognize the importance of contextualizing our methodology in relation to existing studies. To address this, we have included a section in the revised manuscript that succinctly summarizes relevant case studies from other regions and outlines the methodologies they employed. By doing so, we aim to provide readers with a comprehensive understanding of the different approaches used in similar surveillance efforts.  Furthermore, where applicable, we have justified why our chosen methodology is better suited to address the unique challenges or objectives of our study compared to previously employed methods. This serves to underscore the relevance and significance of our research in advancing the field of salt surveillance.  We believe that these revisions enhance the clarity and completeness of our manuscript, and we hope that they address your concerns adequately. Please do not hesitate to reach out if you require any further information or clarification. |
| The editor will carefully examine your introduction, please kindly implement the above to make your introduction very robust |  |
| b) In the materials and methods, study design section needs further elaboration. What is surveillance system evaluation? What does it entail? Define it, explain it, what principles guide it? Just some basic information...please enlighten the readers. | Study design section has been modified, further information has been added. |
| Please, provide a "schematic design pathway of the case study" showing the major stages of the work, how the surveillance evaluation pathways were developed, what comprised the various steps, regional data collection, district data collection, which techniques were employed and at which stage? Please the editor will examine this carefully | The schematic design pathway of the case study" showing the major stages of the work, how the surveillance evaluation pathways were developed has been developed |
|  |  |
| c) Please, provide map of Tanzania, and situate the region surveyed, and further narrowed down to the districts. The rationale of selecting the region, and the subsequent districts appears not sufficient. Please, add more information, provide references to substantiate the selection. Please, add more information as to why government officials are integral to success of the USI surveillance system in Tanzania. Provide adequate references to support this claim. | As you suggested, a map of Tanzania, and situate the region surveyed, and further narrowed down to the districts has been developed. The rationale of selecting the region, and the subsequent districts appears have been clearly explained. more information as to why government officials are integral to success of the USI surveillance system in Tanzania has been clearly elaborated |
| d) Results section appears ok, please kindly merge a couple of sub-sections, given that these subsections are already tabulated. Make results to have maximum of three sub-sections, ok. Discussion section does not need to have subsections, as already shown. | Thank you so much for this important suggestions  Couple of sub-sections have been merged to make three subsection in total |
| e) please, change 'conclusions' to 'concluding remarks' and kindly remove all the sub-headings, and merge them all together, and use paragraphs to different them. | Thank you so much for this important suggestions   - The Word Conclusion has been changed to concluding Remarks. - All sub-headings have been removed and merged. - Paragraphs have been used to different them. |
|  |  |
| Reviewer #1 |  |
| There are few grammar errors, please correct it. | Thank you, the grammatical error was corrected to "The source of data at the national level" |
| National Level Data Collection: The source of data Source at national level - This should be corrected. |  |
| Acceptability: However, apart from Region and District Nutrition Officers - regional | The word Region have been changed to Regional as you suggested, thank you so much |
